# Supplementary material for: Machine learning–based new classification for immune infiltration of gliomas
Source: PLoS One. 2024 Oct 25;19(10):e0312071. doi: 10.1371/journal.pone.0312071 (PMC11508054; doi:10.1371/journal.pone.0312071)
Supplement: Attachment — Submitted filename: Response to Reviewers.docx [file pone.0312071.s006.docx]

Dear Dr. Michael C Burger,

Thank you very much for your time involved in reviewing this manuscript. We have studied your comments carefully and carefully revised the manuscript as suggested.

**Q1.** Please ensure that your manuscript meets PLOS ONE's style requirements, including those for file naming. The PLOS ONE style templates can be found at https:// journals. plos.org/ plosone/s/file?id=wjVg/PLOSOne_formatting_sample_main_body.pdf and https://journals. plos.org/plosone/s/file?id=ba62/PLOSOne_formatting_sample_title_authors_affiliations.pdf.

**Response 1:** Thanks for these suggestions. We referenced the PLOS ONE style template and made the necessary modifications to meet its requirements.

**Q2.** Please state what role the funders took in the study. If the funders had no role, please state: "The funders had no role in study design, data collection and analysis, decision to publish, or preparation of the manuscript." If this statement is not correct you must amend it as needed. Please include this amended Role of Funder statement in your cover letter; we will change the online submission form on your behalf.

**Response 2:** Thanks for these suggestions. We have attached a revised role of funder statement to the cover letter.

**Q3.** We note that you have provided funding information that is not currently declared in your Funding Statement. However, funding information should not appear in the Acknowledgments section or other areas of your manuscript. We will only publish funding information present in the Funding Statement section of the online submission form. Please remove any funding-related text from the manuscript and let us know how you would like to update your Funding Statement. Currently, your Funding Statement reads as follows: "This paper is funded by Anhui provincial key clinical specialties of the 14th Five-Year Plan (2021-25)."

Please include your amended statements within your cover letter; we will change the online submission form on your behalf.

**Response 3:** Thanks for these suggestions. We have made revisions to the acknowledgments section to meet the requirements.

**Q4.** We note that your Data Availability Statement is currently as follows: [All relevant data are within the manuscript and its Supporting Information files.]

Please confirm at this time whether or not your submission contains all raw data required to replicate the results of your study. Authors must share the “minimal data set” for their submission. PLOS defines the minimal data set to consist of the data required to replicate all study findings reported in the article, as well as related metadata and methods (https:// journals. plos.org /plosone /s/data-availability#loc-minimal-data-set-definition).

**Response 4:** Thanks for these suggestions. We have made the modifications as requested.

**Q5.** Please review your reference list to ensure that it is complete and correct. If you have cited papers that have been retracted, please include the rationale for doing so in the manuscript text, or remove these references and replace them with relevant current references. Any changes to the reference list should be mentioned in the rebuttal letter that accompanies your revised manuscript. If you need to cite a retracted article, indicate the article’s retracted status in the References list and also include a citation and full reference for the retraction notice.

**Response 5:** Thanks for these suggestions. We have reviewed all the references in the list, and they meet the required standards.

Dear Reviewer 1,

Thank you very much for your time involved in reviewing this manuscript. We have studied your comments carefully and carefully revised the manuscript as suggested.

**Q1.** Subtypes were ambiguously defined.

Enrichment analysis linked IM1-IMD subtypes with IL-8 and TNF pathways, IM2-IMB with leukocyte activation and NK cell cytotoxicity, IM3-IMC with mitotic processes, and IM4-IMA with CNS and muscle development. These associations are descriptive and lack unified biological significance, let alone clinical relevance.

Clustering analysis from figures demonstrates unclear differences among groups, and survival analyses lack reproducibility.

**Response 1:** Thanks for these suggestions. For the definition of each subtype, we first analyzed the clinical prognosis and immune infiltration characteristics of each subtype; then, based on the differential expression genes (DEGs) of each subtype, enrichment analysis was performed to obtain the biological processes that each subtype may be involved in; finally, the clinical molecular characteristics of each subtype were analyzed. This study defines different subtypes based on prognosis, immune infiltration characteristics, enrichment analysis, and clinical molecular characteristics. At the same time, this study included gliomas from both the CGGA and TCGA databases for mutual verification. Of course, more genomic data analysis and relevant molecular biology experiments will be needed in the future to validate these conclusions. Hope this explanation has addressed your concerns.

Dear Reviewer 2,

Thank you very much for your time involved in reviewing this manuscript. We are very grateful for your evaluation and recognition of this study.

Minor issues:

**Q1.** The manuscript mentions the use of the CGGA and TCGA datasets, but lacks detail on how these datasets were selected, processed, and used in analyses. Please include a detailed description of the datasets, such as the types of data available (e.g., RNA-seq, clinical data), and specific cohorts.

**Response 1:** Thanks for these suggestions. We have added description of the datasets in Materials and Methods as you suggested (Line 171-177). Hope the adjustment has addressed your concern.

**Q2.** Readers may need more information about the normalization methods for the transcriptomic data.

**Response 2:** Thanks for the comment. We have added description of the datasets in Materials and Methods as you suggested (Line 177-179). Hope this response has addressed your concern.

**Q3.** Please consider describing how the differentially expressed genes (DEGs) were selected, including the criteria for differential expression such as statistical thresholds (e.g., p-value, fold change) and the tools used.

**Response 3:** Thanks for this suggestion. We have added description of the selection criteria for DEGs in Materials and Methods as you suggested (Line 186-189). Hope this response has addressed your concern.

**Q4.** The manuscript briefly mentions using DAVID for GO and KEGG pathway analyses without details on the processes involved. Please consider mentioning more of the DAVID tool overview and specific settings applied.

**Response 4:** Thanks for this suggestion. We have added description of the DAVID tool in Materials and Methods as you suggested (Line 196-203). Hope this response has addressed your concern.

**Q5.** In the part of the discussion related to proposed strategies for different immune subtypes, it would be beneficial to directly refer to the relevant medical literature to stronger justify the treatment recommendations.

**Response 5:** Thanks for this constructive comment. This detailed description was added to Line 387-402. Hope this response has addressed your concern.

**Q6.** Please acknowledge the study limitations and suggest future research directions.

**Response 6:** Thanks for this valuable suggestion. This detailed description was added to Line 421-425. Hope the adjustment has addressed your concern.

Again, we highly appreciate your comments for this manuscript and wish these explanations and corrections have addressed your concerns.
